# Investigating the therapeutic potential of the antibacterial drug nitroxoline in combating cryptococcal infection

**DOI:** 10.1371/journal.pntd.0014627

**Published:** 2026-08-17

**Authors:** Nour M. Alkashef, Ammar A. Khan, Mohamed N. Seleem

**Affiliations:** 1 Department of Biomedical Sciences and Pathobiology, Virginia-Maryland College of Veterinary Medicine, Virginia Polytechnic Institute and State University, Blacksburg, Virginia, United States of America; 2 Center for One Health Research, Virginia Polytechnic Institute and State University, Blacksburg, Virginia, United States of America; Faculdades Pequeno Príncipe: Faculdades Pequeno Principe, BRAZIL

## Abstract

Cryptococcosis is a major health threat among immunocompromised individuals, resulting in life-threatening consequences secondary to brain invasion. The limited treatment options and the increased incidence of treatment failure and relapses urge the need for more effective therapies. In this study, we investigated the therapeutic potential of nitroxoline (NTX) in treating cryptococcal infection. NTX exhibited potent fungicidal activity against *Cryptococcus neoformans/gattii* clinical isolates, with an MIC_90_ of 1 µg/mL. In addition, NTX substantially inhibits the main virulence traits of cryptococcal cells, including the polysaccharide capsule, melanin production, and the urease enzyme, which are crucial for the infection progression. In exploring the potential mechanism of action, our results revealed the relationship between the metal-chelating properties of the NTX scaffold and its antifungal activity. Interfering metal chelation, either through structural modification or exogenous supplementation with varied divalent cations, significantly reduced the antifungal and anti-virulence activities of NTX. Given its potential therapeutic role, we evaluated the interaction between NTX and the clinically used antifungals amphotericin B, flucytosine and fluconazole. Additionally, we investigated the tendency of cryptococcal cells to develop resistance following repeated exposure to NTX, as well as its efficacy in the *Caenorhabditis elegans* nematode model of cryptococcal infection. Notably, cryptococcal cells exhibited consistent susceptibility to NTX upon repeated exposure to a subinhibitory concentration. In the *C. elegans* model, NTX significantly reduced the fungal burden of the infected worms, achieving a 2-log10 reduction, and consistently enhanced their survival compared to untreated group. These findings highlight the potential role of the NTX scaffold in combating cryptococcal infection that warrants further investigation.

## Author summary

Nitroxoline (NTX) antibiotic has been prescribed for decades to treat acute and recurrent bacterial urinary tract infections. Its extensive urinary excretion has limited its therapeutic utility for invasive bacterial infections. However, its favorable safety profile, excellent oral bioavailability, and the low potential of resistance, have encouraged its repurposing against a diverse range of pathogens. In this study, we investigated NTX activity against *Cryptococcus* fungal pathogen that can disseminate to the central nervous system, leading to fatal meningoencephalitis in immunocompromised patients. The mortality rate of this opportunistic infection can exceed 60% especially in resource-limited regions. NTX exhibited potent fungicidal activity against proliferated cryptococcal cells, with an inhibitory activity on the main virulence factors involved in the initial colonization, including capsule formation, melanin pigmentation and urease enzyme. This anti-virulence activity is directly related to the metal chelation nature of the NTX scaffold, enabling the depletion of the intracellular content of essential cations. Furthermore, cryptococcal cells exhibited a negligible resistance tendency during continuous exposure to NTX. Overall, these findings demonstrate that NTX exerts both direct antifungal and anti-virulence activities on *Cryptococcus* through a metal chelation-dependent mechanism and highlight its potential role as a repurposed therapeutic for the treatment of cryptococcosis.

## Introduction

Cryptococcosis is an opportunistic fungal infection that primarily affects individuals with compromised immune system. This invasive infection begins by inhaling the fungal spores of the *C. neoformans/gattii* species, which colonize the alveolar space, establishing an active infection or remaining dormant depending on the competence of the immune system [1,2]. The invading cryptococcal cells can then disseminate towards the central nervous system, resulting in life-threatening meningitis [3,4]. Annually, cryptococcal infections affect approximately 194,000 immunocompromised individuals worldwide, primarily HIV/AIDS patients, with a mortality rate exceeding 75.8%, especially in low to middle-income regions [5,6].

The intrinsic resistance of cryptococcal cells to echinocandins limits the current therapeutic arsenal to three systemic antifungals: amphotericin B (AmB), fluconazole (FLC), and flucytosine (5-FC) [7,8]. Current clinical guidelines predominantly rely on the combination of AmB and 5-FC to elicit early fungal clearance during the induction stage of treatment [9,10]. On the other hand, FLC has been proven to be effective in preventing relapses during both consolidation and maintenance stages of treatment [9]. However, the scarcity of the first-line antifungals, AmB and 5-FC, in resource-limited regions and their related toxicities, requiring continuous treatment monitoring, make it challenging to overcome the high fatality and escalating relapses of this infection with alternative FLC monotherapy, even with higher doses up to 1200 mg/daily [11–13].

Recent efforts to reposition approved drugs for cryptococcal infections have efficiently replenished the therapeutic pipeline with a variety of candidate molecules [14,15]. Previously, we identified the antifungal activity of the 5-nitro-8-hydroxyquinoline scaffold, nitroxoline (NTX), against the *Cryptococcus* pathogen through screening of 3,700 therapeutic molecules, including FDA-approved drugs and investigational molecules [16]. Over the past 50 years, NTX has been used primarily in combating acute and recurrent infections caused by a broad spectrum of urinary tract pathogens [17]. This antibacterial activity is considered to be related to its ability to chelate essential metals needed for the activity of bacterial RNA polymerase enzymes [18]. Its favorable safety profile and superior oral bioavailability have encouraged its repurpose against a diverse range of bacterial, viral, and parasitic pathogens [19–23]. However, its short plasma half-life and extensive urinary excretion have limited its clinical usage to urinary infections [24].

In this study, we investigated the therapeutic potential of NTX against the *Cryptococcus* yeast. NTX demonstrated potent antifungal activity against the tested cryptococcal isolates, with a fungicidal activity on proliferating cryptococcal cells. NTX also demonstrated potent inhibitory activity against the key virulence factors of cryptococcal cells involved in the alveolar colonization and dissemination. Additionally, we investigated the relationship between the metal chelation activity of the NTX scaffold and its antifungal properties. Most importantly, the tendency of cryptococcal cells to develop resistance to NTX upon repeated exposure was investigated to highlight its therapeutic potential. The efficacy of NTX was also evaluated in the *Caenorhabditis elegans* nematode model of cryptococcal infection.

## Materials and methods

### Strains, drugs, and chemicals

Clinical isolates of the *C. neoformans/gattii* complex were obtained from the American Type Culture Collection, ATCC (Manassas, VA, USA) and BEI Resources (Manassas, VA, USA). Prior to any experiment, fungal isolates were recovered from 20% glycerol stocks stored at −80°C on YPD agar (Becton, Dickinson and Company, MD, USA). Tested drugs including NTX (Cayman chemicals, MI, USA), NTX analogs (Mcule, CA, USA) and standard antifungals, including AmB (Chem-Impex International, IL, USA), FLC (Thermo Fisher, NC, USA), and 5-FC (Thermo Scientific, MA, USA), were solubilized in DMSO (Thermo Fisher, NC, USA) and stored at -20 °C. RPMI 1640 medium (Gibco, NY, USA) was prepared according to the manufacturer’s guidelines and was buffered with 3-(N-morpholino)propanesulfonic acid (Fisher Bioreagents, NJ, USA). Metal cations: CuSO_4_, ZnCl_2,_ MgCl_2_, CaCl_2_, FeCl_2,_ and MnCl_2_ (Sigma-Aldrich, MO, USA) were dissolved in sterilized double-distilled H_2_O.

### In vitro evaluation of the antifungal activity

The minimum inhibitory concentration (MIC) for tested compounds and standard antifungals was determined using the standard microdilution technique following the Clinical and Laboratory Standards Institute (CLSI) guidelines M27-A3 for yeast [25]. Cryptococcal cells from overnight cultures in YPD broth were diluted in RPMI-MOPS medium to achieve a final density of 1 × 10^3^ CFU/mL. The MIC was assessed after 72 hours of incubation at 35 °C. Complete growth inhibition, compared to the untreated culture, was used as the endpoint for both NTX and AmB, while a 50% reduction in fungal growth was considered the endpoint for fluconazole and 5-FC.

### Microdilution checkerboard assay

The impact of combining NTX with standard antifungals, including AmB and FLC, was evaluated using the microdilution checkerboard method as previously described [16,26]. The efficacy of varied combinations was evaluated after incubation at 35 °C for 72 hrs. The optical density was measured at 540 nm and expressed relative to the growth of the untreated inoculum. The fractional inhibitory concentration index (ΣFICI) was calculated to interpret the interaction between combined drugs. Combined drugs were considered to interact synergistically if FICI values were ≤ 0.5. On the other hand, FICI values > 0.5 and 1 indicate additive interaction, whereas values between 1 and 2 or > 4 indicate indifferent and antagonistic interaction, respectively [27].

### Time-kill assay

The impact of NTX on the growth kinetics of Cryptococcal cells was evaluated as previously described [28]. Logarithmically growing cells from an overnight culture of *C. neoformans* H99 in YPD broth were collected and adjusted to ~ 5 × 10^3^ CFU/mL in RPMI-MOPS medium. Equal aliquots were treated with different concentrations of NTX and AmB, as standard antifungal, and were incubated at 35 °C for 72 hours. The number of viable cells was monitored at various points (0, 2, 4, 6, 12, 24, 48, and 72 hrs) by 1:10 diluting with PBS and platting on YPD agar.

### Anti-virulence characterization

#### Urease activity.

Cells from an overnight culture of *C. neoformans* H99 strains were adjusted to a final density of ~1 × 10^4^ CFU/mL in urease broth and incubated in the presence of sublethal concentrations of NTX (up to 0.5x MIC), alongside acetohydroxamic acid (3.33 mM) as a positive control, at 30 °C for 72 hours. Following the incubation period, cells were removed by centrifugation at 10,000 rpm for 5 minutes, and the absorbance of the cell-free aliquots was measured at 560 nm [29,30].

#### Induction of Melanin pigmentation.

The induction of melanin production was evaluated using liquid minimal medium (15 mM glucose, 10 mM MgSO_4_, 29.4 mM KH_2_PO_4_, 13 mM glycine, 3 µM thiamine) supplemented with 1 mM L-DOPA as previously described [29,31]. Minimal medium was inoculated with *C. neoformans* H99 strain (~1 × 10^4^ CFU/mL) and equal aliquots were dispensed into 24-well microtiter plates in the presence of subinhibitory concentrations of NTX (up to 0.5x MIC), alongside 5-FC as a positive control. Plates were incubated in the dark at 30 °C and were monitored for 10 days. The mean gray value of the treated cultures was quantified in grayscale using ImageJ software as previously described [32,33].

#### Capsule size analysis.

Capsule formation was induced in Dulbecco’s Modified Eagle Medium (DMEM) supplemented with 10% fetal bovine serum (FBS) at 37°C and 5% CO_2_ for 3 days in the presence of a sublethal concentration of NTX (0.5x MIC). AmB (0.0156 µg/mL) was included as a positive control. Cells were collected by centrifugation at 10,000 rpm for 5 minutes and were subjected to microscopic analysis using India ink (Thermo Scientific, MA, USA). Measurement of the whole cell diameter and cell body diameter was conducted using ImageJ software. The impact of varied treatments on capsule thickness was assessed for 50 cells from each treatment by subtracting the cell body diameter from the entire cell diameter [34–36].

### PGFL assay

The effect of NTX treatment on the intracellular content of metal cations was evaluated using the fluorescent probe PhenGreen FL diacetate (PGFL), as previously described [37]. Logarithmically grown *C. neoformans* H99 were adjusted to ~1 × 10^6^ CFU/mL in RPMI-MOPS medium and were treated with varying concentrations of NTX (up to 0.5 µg/mL) for 6 hours at 30°C. Cells were rinsed and incubated with 2.5 µM PGFL (Invitrogen, CA, USA) for 30 minutes. Following the incubation time, cells were rinsed again for three times with distilled water and transferred to an opaque 96-well microtiter plate. The fluorescence intensity was measured at excitation and emission wavelengths of 490 nm and 520 nm, respectively.

### Drug resistance analysis

The tendency of cryptococcal cells to adapt to the activity of NTX was evaluated using frequent passaging as previously described [16,38,39]. *C. neoformans* H99 cells (~1x10^5^ CFU/mL) in RPMI-MOPS medium were treated with a 0.5x MIC of the tested compounds, including 5-FC (2 μg/mL) as a positive control, for 48 hrs at 35°C. Treated cells were collected by centrifugation at 10,000 rpm for 5 minutes and were adjusted to the same density for the following passage. Frequent passaging of cryptococcal cells was continued for 20 passages, and the susceptibility of treated cells was evaluated after each passage.

### The nematode model of Caenorhabditis elegans

The efficacy of NTX was evaluated in the *C. elegans* model as previously described [28,40]. Worms of *C*. *elegans* AU37 (*Δglp-4; Δsek-1*) were synchronized and allowed to reach L4 larval stage on a lawn of *Escherichia coli* OP50 strain on standard nematode growth medium [41]. Synchronized larva were then infected with *C*. *neoformans* H99 (~1x10^6^ CFU/mL) in M9 buffer, supplemented with 50 μg/ml of gentamicin to inhibit the growth of *E. coli* OP50, for 6 hrs at 30 °C. Infected worms (300 worm/mL) were rinsed 5 times with an equal volume of M9 buffer and treated with different concentrations of NTX for 24 hrs at 30 °C. Treated worms were then rinsed, resuspended in M9 buffer, and were homogenized with 0.5 mm silicon/zirconia beads (BioSpec Products, OK, USA) using Next advance homogenizer (Next Advance, NY, USA) at 4800 cycle/minute for 6 minutes at room temperature. The fungal burden of differently treated worms was evaluated by diluting and plating their homogenate on gentamicin supplemented YPD agar (100 μg/mL). The survival of treated worms was also monitored for 10 days and data was presented using a Kaplan-Meier survival curve.

### Statistical analysis

The statistical significance of the collected data was assessed by one-way analysis of variance (ANOVA) test for multiple comparisons and log-rank test for Kaplan-Meier survival curve using GraphPad Prism 8.0 Software (La Jolla, CA, USA). Data sets with *p* values of ≤ 0.05 were considered statistically significant.

## Results

### Validating the antifungal activity of NTX against *Cryptococcus* yeast

**Fig 1:**
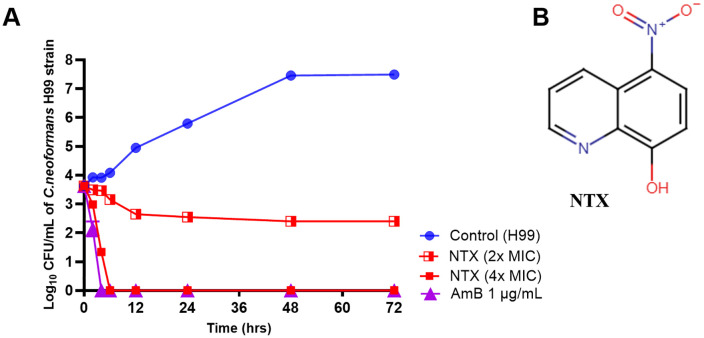
Validating the antifungal activity of NTX on the *Cryptococcus neoformans* pathogen. **(A)** Dose-dependent effect of NTX on the growth kinetics of treated cryptococcal cells. Aliquots of H99 cultures (~5 × 10^3^ CFU/mL) in RPMI-MOPS medium were treated with NTX at different concentrations (up to 4×  MIC), along with AmB (1 µg/mL) as a control antifungal and DMSO (0.2%). Cell proliferation was monitored at the designated time points over 72 hours. The Lower limit of detection (LOD) was 1 CFU/mL, determined by platting 1 ml of treated aliquots on YPD agar. **(B)** Chemical structure of 5-nitro-8-hydroxyquinoline (NTX).

The activity of NTX was tested on a panel of 16 clinical isolates representing the *C. neoformans/gattii* complex. NTX exhibited potent inhibitory activity against the tested isolates, with an MIC_90_ of 1 µg/mL (Table 1). Additionally, the impact of NTX on the growth kinetics of cryptococcal cells was evaluated for 72 hours. NTX exerted concentration-dependent fungicidal activity (Fig 1A), depleting the entire population of the *C. neoformans* H99 strain within 6 hrs at 4x MIC.

**Table 1:** Susceptibility of *C. neoformans/gattii* clinical isolates to nitroxoline and standard antifungals.

Isolate	Description	MIC (µg/mL)			
		NTX	FLC	5-FC	AmB
*C. neoformans* H99 ATCC 208821	Isolate from patient with Hodgkin’s disease, USA.	0.5	2	4	0.25
*C. neoformans* NR-41291	Isolate from human cerebrospinal fluid, China.	1	4	0.5	0.5
*C. neoformans* NR-41292	Isolate from human cerebrospinal fluid, China.	0.25	8	0.5	1
*C. neoformans* NR-41294	Isolate from human cerebrospinal fluid, China.	2	4	2	1
*C. neoformans* NR-41295	Isolate from human cerebrospinal fluid, China.	1	16	0.5	0.5
*C. neoformans* NR-41296	Isolate from human cerebrospinal fluid, China.	0.5	4	0.5	1
*C. neoformans* NR-41297	Isolate from human cerebrospinal fluid, China.	0.5	2	4	0.5
*C. neoformans* NR-41298	Isolate from human cerebrospinal fluid, China.	0.5	4	2	1
*C. neoformans* NR-41299	Isolate from human cerebrospinal fluid, China.	0.25	4	2	1
*C. neoformans* NR-41300	Isolate from human cerebrospinal fluid, China.	0.5	1	1	1
*C. gattii* R265 NR-43208	Isolate from a human during the Vancouver outbreak, Canada	0.25	4	1	1
*C. gattii* CBS1930 NR-43209	Isolate from a goat prior to Vancouver outbreak, Canada.	0.25	8	2	1
*C. gattii* NR-50423	Isolate from human cerebrospinal fluid, Pacific Northwest region of North America.	0.25	8	2	1
*C. gattii* NR-50426	Isolate from human, Pacific Northwest region of North America.	0.25	4	2	1
*C. gattii* NR-50427	Isolate from human, Pacific Northwest region of North America.	0.5	4	2	0.5
*C. gattii* NR-50429	Isolate from human lung biopsy, Pacific Northwest region of North America.	0.25	2	2	0.5

### NTX exhibits potent anti-virulence activity on cryptococcal cells

**Fig 2:**
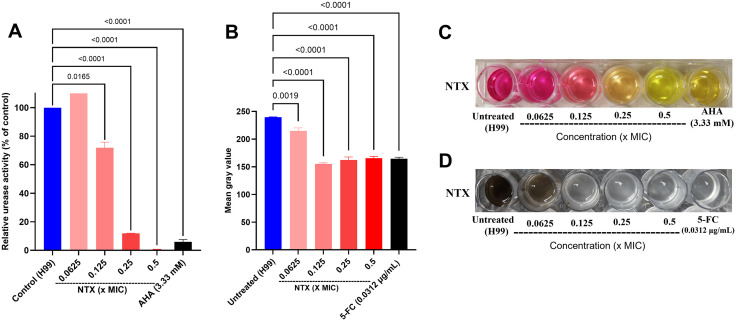
The impact of NTX on urease enzyme activity and melanin pigmentation of cryptococcal cells. **(A)** Urease activity of *C. neoformans* H99 cultures treated with different subinhibitory concentrations of NTX (up to 0.5x MIC) and DMSO (0.25%). Acetohydroxamic acid (AHA) was also included as a positive control at a concentration of 3.33 mM. The color intensity was measured at 560 nm and expressed relative to untreated cultures. Data represent the average of two independent experiments (5 replicates each), relative to the untreated cells. **(B)** Mean gray values of *C. neoformans* H99 cultures induced for melanin production in the presence of varied sublethal concentrations of NTX (up to 0.5x MIC), alongside 5-FC (0.0312 μg/mL) as a positive control and DMSO (0.25%). Data represent the average of two independent experiments. **(C)** Colored photograph for cell-free aliquots of *C. neoformans* H99 cultures in urea broth, in the presence of varied concentrations of NTX and AHA as a positive urease inhibitor. **(D)** Colored photograph for *C. neoformans* H99 in liquid minimal medium supplemented with L-dopa for 10 days at 30 °C, in the presence of different concentrations of NTX and 5-FC as a positive inhibitor for melanin pigmentation. Data was analyzed using one-way analysis of variance (ANOVA) for multiple comparison, and *p* values of ≤ 0.05 indicate statistical significance of compared data sets.

We examined the impact of NTX on the key virulence traits of cryptococcal cells, including urease activity, melanin pigmentation, and capsule formation. Cryptococcal cells exhibited reduced urease activity when grown in urea-containing broth in the presence of sublethal levels of NTX (Fig 2A and 2C). Similarly, NTX significantly impaired the oxidation of L-DOPA to the dark melanin pigment, resulting in non-melanized cultures, even at low concentrations (Fig 2B and 2D).

**Fig 3:**
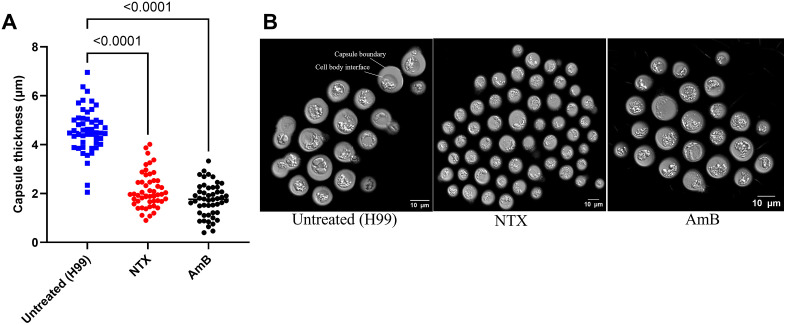
Impact of NTX on the capsule formation of cryptococcal cells. **(A)** Capsule size measurement of cryptococcal cells grown in Dulbecco’s modified Eagle medium supplemented with 10% fetal bovine serum (FBS) in the presence of subinhibitory concentration of NTX (0.5x MIC), AmB (0.0156 µg/mL) as a positive control, or DMSO (0.2%). Data represent the average measurement of 50 cells from each individually treated aliquots of *C. neoformans* H99 strain. **(B)** Corresponding light microscopy images for individually treated aliquots stained with India ink (1:1). Data was analyzed using one-way analysis of variance (ANOVA) for multiple comparison, and *p* values of ≤ 0.05 indicate statistical significance of compared data sets.

Additionally, we investigated the impact of NTX on the formation of capsular polysaccharide in cryptococcal cells grown under induction conditions. Cryptococcal cells treated with a sublethal concentration of NTX (0.5x MIC) produced significantly smaller capsules, with an average thickness of 2.13 ± 0.72 µm. On the other hand, untreated cryptococcal cells grown under the same conditions produced capsules with an average thickness of 4.59 ± 0.89 µm (Fig 3A and 3B).

### Metal chelation is a major contributor controlling NTX activity

**Fig 4:**
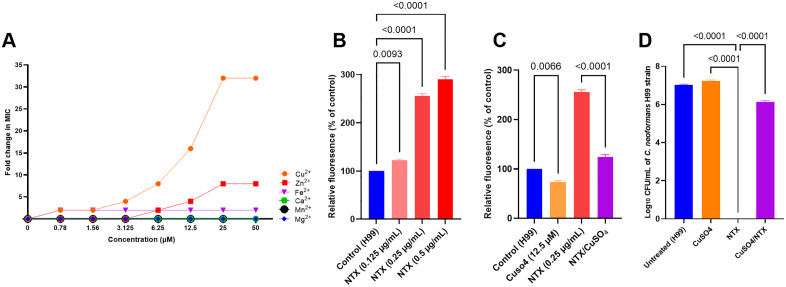
Impact of NTX on the metal homeostasis of cryptococcal cells. **(A)** The impact of divalent cation supplementation on the antifungal activity of NTX. The change in the MIC was evaluated in RPMI-MOPS medium supplemented with increasing concentrations of various divalent cations (up to 50 μM). **(B)** PGFL assay for the intracellular metal content of cryptococcal cells (~1 × 10^6^ CFU/mL) treated with varied concentrations of NTX or DMSO (0.5%). **(C)** Effect of CuSO4 supplementation (12.5 µM) on the intracellular metal content of cryptococcal cells treated with NTX (0.25 µg/mL) and DMSO (0.25%). Data represent the average of two independent experiment relative to the untreated cells. **(D)** Effect of CuSO4 supplementation (12.5 µM) on the growth of *C. neoformans* H99 (~ 5 × 10^3^ CFU/mL) in RPMI-MOPS medium after 72 hrs in the presence of 4x MIC of NTX (2 µg/mL) or DMSO (0.2%). Data was analyzed using one-way analysis of variance (ANOVA) for multiple comparison, and *p* values of ≤ 0.05 indicate statistical significance of compared data sets.

Initially, we tested whether exogenous supplementation with various divalent cations would affect the antifungal activity of NTX. Supplementing RPMI-MOPS medium with increasing concentrations of copper ions significantly impaired NTX activity, resulting in a 32-fold increase in the MIC at 25 µM. Supplementation with Zn^2+^ and Fe^2+^ had a similar effect on NTX activity, resulting in 8- and 2-fold increases in its MIC, respectively. In contrast, the susceptibility of cryptococcal cells to NTX remained unchanged in the presence of other divalent cations, including Ca^2+^, Mn^2+^, and Mg^2+^, highlighting the metal preference of the NTX molecule (Fig 4A).

Secondly, we utilized the PhenGreen FL diacetate (PGFL) fluorescent probe to gain direct insight into the intracellular metal content of cryptococcal cells in response to NTX treatment. Cryptococcal cells demonstrated a significant dose-dependent increase in the PGFL fluorescence following exposure to different concentrations of NTX, indicating the stripping of essential metal cations (Fig 4B). This fluorescence upshift was significantly reversed upon supplementation with CuSO_4_ (12.5 µM), resulting in the quenching of PGFL in the presence of excess metal (Fig 4C). Consistently, supplementing RPMI-MOPS medium with CuSO_4_ (12.5 µM) rescued cryptococcal cells treated with NTX at 4x MIC (Fig 4D).

**Fig 5:**
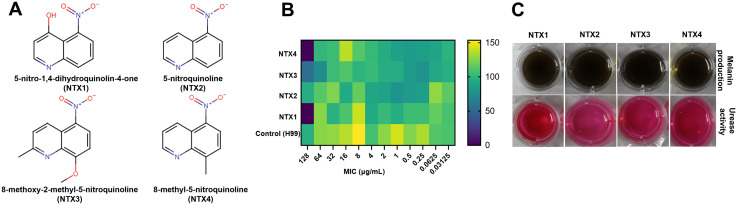
Relationship between metal chelation properties of NTX scaffold and its antifungal activity. **(A)** Chemical structures of NTX analogs with modified chelation center. **(B)** Antifungal activity of the tested NTX analogs on *C. neoformans* H99 strain. OD was measured at 540 nm, and the growth intensity was presented relative to the control growth as a heat map. **(C)** Anti-virulence activity of tested NTX analogs. The upper and lower panels show the effects of the tested analogs on melanin production and urease enzyme activity of cryptococcal cells treated with 0.5x MIC of each analog.

Furthermore, we tested whether the loss of the metal-chelating center would affect the antifungal activity of the NTX scaffold. All 4 tested analogs with disabled chelating activity due to the absence of the electron-donating 8-hydroxyl group within the chelation center of the NTX scaffold (Fig 5A), including 5-nitro-1,4-dihydroquinolin-4-one, 5-nitroquinoline, 8-methoxy-2-methyl-5-nitroquinoline, and 8-methyl-5-nitroquinoline, did not show antifungal activity against cryptococcal cells up to 64 µg/mL (Fig 5B). Similarly, these tested analogs did not affect the activity of urease enzyme or melanin pigmentation of treated cryptococcal cells (Fig 5C), highlighting the relationship between metal chelation and the antifungal attributes of NTX scaffold.

### Drug interaction with standard antifungals

**Fig 6:**
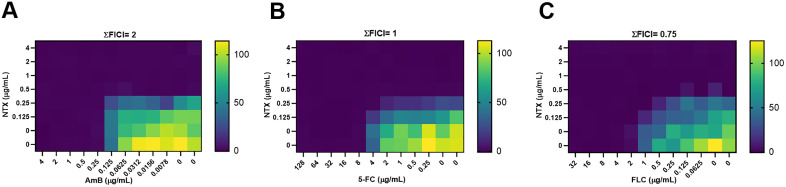
Interaction between NTX and components of current treatment regimen. The efficacy of the combination between NTX (0.125-4 μg/mL) and either AmB (0.0078-4 μg/mL), 5-FC (0.25-128 μg/mL), or FLC (0.0625-32 μg/mL) was evaluated on *C. neoformans* H99 strain using checkerboard microdilution. Following incubation for 72 hrs at 30°C, the optical density (OD) was measured at 540 nm, and growth intensity was presented relative to the control growth as heat maps. The fractional inhibitory concentration index (ΣFICI) was used to interpret the outcome combined drugs where FICI ≤ 0.5 or between > 0.5 and 1 indicate synergistic and additive interaction, respectively. FICI values between 1 and 2, or > 4 indicate both indifferent and antagonistic interactions, respectively.

The interaction between NTX and clinically used antifungals, including parenteral AmB and oral 5-FC and FLC, was evaluated using a microdilution checkerboard assay. Combining NTX with AmB showed indifferent interaction (FICI = 2) with no change in the individual MICs of both combined drugs (Fig 6A). On the other hand, NTX exhibited enhanced activity when combined with either 5-FC or FLC, resulting in an additive interaction with a FICI of 1 and 0.75, respectively (Fig 6B and 6C), Which highlights the beneficial outcome of these combinations.

### Evaluation of resistance development to NTX treatment during serial passaging

**Fig 7:**
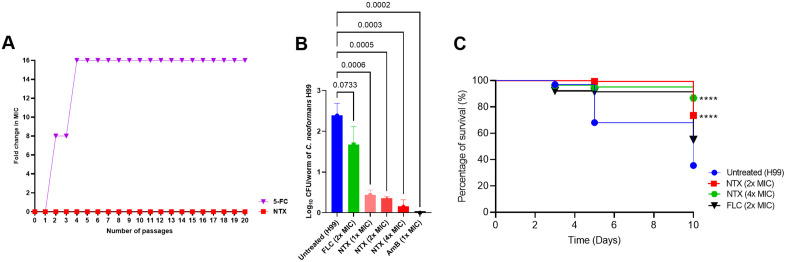
Evaluation of the resistance tendency of cryptococcal cells and the efficacy of NTX in the *Caenorhabditis elegans* nematode model. **A)** Resistance-tendency analysis of cryptococcal cells to NTX. Aliquots of C. neoformans H99 strain in RPMI-MOPS (~1 × 10^5^ CFU/mL) were continuously exposed to a subinhibitory concentration (0.5 MIC) of NTX, along with 5-FC (2 μg/mL) as a positive control. Cells from individually treated cultures were evaluated for their susceptibility following each passage, and the fold change in MIC was plotted versus the corresponding passage number. **(B)** Efficacy of NTX in the *C. elegans* model. Infected L4 larvae (300 worms per mL) were individually treated with NTX (up to 4x MIC), along with AmB and FLC as positive controls and DMSO (0.4%), for 24 hrs at 30°C. Treated worms were homogenized and plated onto gentamicin-supplemented YPD agar to assess the change in fungal burden. Data represent the average of two independent experiments (4 replicates each). Data was analyzed using one-way analysis of variance (ANOVA) for multiple comparison, and *p* values of ≤ 0.05 indicate statistical significance of compared data sets. **(C)** A Kaplan-Meier survival curve of *C. elegans* worm groups treated with NTX (up to 4x MIC), FLC (2x MIC) as a positive control or DMSO (0.4%). Statistical significance was evaluated using log-rank test, and **** indicates statistical significance at *p* value of ≤ 0.0001.

The tendency of cryptococcal cells to develop resistance to NTX was evaluated by repeated exposure to a sublethal concentration (0.5 × MIC) of NTX, along with 5-FC as a positive control. Notably, cryptococcal cells showed consistent susceptibility to NTX with no change in initial MIC (0.5 μg/mL) over 20 consecutive passages (Fig 7A). In contrast, cells treated with a sublethal concentration of 5-FC showed a 16-fold increase in the initial MIC (4 μg/mL) during the first four passages.

### Efficacy of NTX in the *Caenorhabditis elegans* model of cryptococcal infection

The efficacy of NTX was further assessed in the *C. elegans* model, alongside the standard antifungals AmB and FLC. NTX treatment significantly reduced the fungal burden of the treated worms. Following treatment for 24 hours, NTX treatment at different concentrations (up to 4x MIC) achieved ~2 log10 reduction in the colony counting of homogenized worms compared with the untreated worms (Fig 7B). Similarly, AmB treatment depleted the fungal population within the treated worms, achieving ~ 2.35 log10 reduction compared with untreated worms. On the other hand, FLC (2x MIC) didn’t achieve a significant reduction in the fungal burden of treated worms (-0.71 log_10_). Next, we assessed whether NTX treatment would affect the survival of *C. elegans* worms infected with *C. neoformans* H99 (Fig 7C). At 10 days postinfection, the untreated larval group showed a survival rate of ~35.6% while groups treated with NTX at 2x MIC and 4x MIC exhibited a significantly enhanced survival rate of ~ 73.5% and 86.6% (*p* < 0.0001), respectively.

Additionally, we evaluated the impact of combination between FLC and NTX on the fungal burden and the survival of infected worms. Compared to untreated worm group, combining NTX at a sublethal level (0.5x MIC) with FLC (2x MIC) resulted in ~ 0.9 log10 reduction (S1A Fig). On the other hand, FLC/NTX combination didn’t affect the survival of treated worm groups (S1B Fig).

## Discussion

*Cryptococcus* pathogen is the leading cause of fungal meningoencephalitis among immunocompromised individuals, accounting for 19% of HIV-related mortalities [5,6]. The treatment outcome of this invasive infection mainly relies on achieving early fungal clearance, particularly within the brain parenchyma and CSF [42]. The pharmacodynamic properties of the used antifungals and their dynamics within targeted body compartments are the main factors affecting treatment outcomes [43]. Combining intravenous AmB with oral 5-FC is the optimal treatment for CM infection due to its fungicidal activity and efficient penetration to the brain compartments [42,44]. However, limited access to these antifungals and the need for continuous treatment monitoring, especially with hepatic and renal comorbidities, urge the alternative use of FLC which results in treatment failure and relapses due to its fungistatic nature [7,45]. Hence, current clinical outcomes for cryptococcal infections, especially in resource-limited regions, underscore the need for more effective antifungals with improved tolerability and wider availability.

Repurposing drugs with established pharmacokinetic and safety profiles provides a fast track toward potentially effective antifungals, avoiding substantial costs related to preclinical development [46]. Structural optimization can further expedite the development of candidate entities by enhancing their efficacy, selectivity, and distribution within the targeted body compartments [47]. In this study, we identified the antifungal activity of NTX against *Cryptococcus* pathogen, with a MIC_90_ of 1 µg/mL on the tested panel of clinical isolates. In the time-kill assay, NTX exhibited potent fungicidal activity on proliferating cryptococcal cells, which is a notable advantage for candidate molecules targeting cryptococcal infections. Consistently, NTX has been reported to be active against other superficial and invasive fungal pathogens, including azole-resistant *Aspergillus* and multidrug-resistant *Candida spp.* [48–50], highlighting the spectrum of its antifungal activity.

Targeting the key virulence traits of cryptococcal cells is another advantage for NTX, further supporting its use during the consolidation and maintenance stages of treatment, in combination with oral FLC [51]. Among the existing antifungal arsenal, only 5-FC exhibits anti-virulence activity on cryptococcal cells by inhibiting melanin production, which may contribute to its efficacy when combined with AmB at the early treatment stage [31]. The survival of the cryptococcal cells within the alveolar space mainly depends on the protective function of the capsular polysaccharide and melanin [52,53]. On the other hand, the activity of the urease enzyme is crucial for breaching the BBB endothelium, thereby allowing brain invasion [54,55]. Hence, disrupting the function of these traits could significantly impact the outcome of cryptococcal infection.

The ability of NTX to inhibit these two metallo-centric enzymes, laccase and urease, is primarily related to the metal-chelating activity of the nitrogen atom of the quinoline ring and the 8-hydroxyl moiety of this scaffold [56,57]. This anti-virulence trait aligns with the reported activity of NTX on metallo-β-lactamase-producing pathogens through the chelation of zinc [57–59]. Cryptococcal laccase enzyme utilizes copper ions as a cofactor to oxidize norepinephrine and other abundant catecholamines within the brain parenchyma into dark melanin pigmentation [60,61]. The limited level of copper metal within the brain and its essentiality for the cerebral progression of cryptococcal infection highlights the potential role of NTX in halting cryptococcal growth within the brain parenchyma by depriving the invading cells of copper [62]. Similarly, inhibiting the activity of the urease enzyme by targeting nickel ions within the enzyme complex could significantly impede the transmigration of cryptococcal cells toward the brain [55].

While there is debate over whether NTX could affect other molecular targets, our results indicate that the antifungal activity of the NTX scaffold is directly related to metal chelation, which significantly affects various cellular activities necessary for pathogen survival [63,64]. Similar to other cell-permeable metal chelators, NTX treatment resulted in a dose-dependent increase in PGFL fluorescence, indicating depletion of essential metals within cryptococcal cells [65]. Consistently, NTX was reported to affect metal homeostasis in *Acanthamoeba castellanii* amoebae and *A. fumigatus*, leading to the depletion of iron and copper metals, respectively [66,67]. The impact of exogenous supplementation with various divalent cations, particularly Cu^2+^ and Zn^2+^, on NTX activity also supports metal homeostasis as the primary target of NTX in cryptococcal cells. Notably, NTX metal preference varies among other pathogens. While Mn^2+^ and Mg^2+^ significantly affect the antibacterial activity of NTX against *E. coli*, supplementation with these two cations doesn’t exert any change on the MIC of NTX against cryptococcal cells [56]. Additionally, the loss of both lethality and anti-virulence activity with tested NTX analogs, secondary to the substitution of 8-hydroxyl moiety at the chelation center, underscores the crucial link between metal chelation and the activity of the NTX scaffold against cryptococcal cells.

The beneficial interaction between NTX and standard antifungals, especially oral FLC, highlights the potential role of NTX in addressing frequent relapses and treatment failures related to FLC monotherapy [68]. FLC is often the only treatment option for cryptococcal infection even during the induction stage treatment due to the restricted access to AmB and 5-FC in resource-limited regions [69,70]. Even at higher doses (800–1200 mg/day), FLC monotherapy showed frequent treatment failure and relapses with mortality rate exceeding 70% within the first year comparing to AmB based regimens [13,71–73]. Hence, combining FLC and NTX could leverage the efficiency of FLC monotherapy considering the fungicidal activity and the anti-virulence advantage of NTX scaffold. Additionally, the low propensity of cryptococcal cells to develop resistance to NTX during continuous exposure, under the used in vitro conditions, also supports its potential role during treatment stages, which needs further validation considering the extended length of the cryptococcal infection treatment and other host related factors.

The pharmacokinetic prosperities of the NTX scaffold pose a significant challenge to its clinical utility against invasive fungal infection [24]. While the oral dosage of NTX achieves adequate plasma level (~ 5.5 µg/mL), its extensive hepatic degradation and excretion via the urinary tract (> 60% of administered dose) limit its systemic exposure [24,74,75]. Despite the scarce data regarding its level within cerebral and pulmonary compartments, reported NTX efficiency in slowing glioma proliferation in mice and resolving granulomatous amebic encephalitis in human*,* in combination with the standard treatment regimen, highlights its ability to reach brain parenchyma and its potential role in combating the cerebral progression of cryptococcal infection [76,77]. Additionally, recent derivatives of the NTX scaffold, including ASN-1733 and ASN-1213, could exhibit superior therapeutic efficacy against cryptococcal infection. Their metabolic stability could allow better systemic exposure for the targeted organs, brain and lung, considering their plasma level of ~ 52.36 and 20 µg/mL and their extended half-life of ~ 22 hrs. and 14.3 hrs., respectively [78]. Moreover, direct drug delivery via inhalation can provide an alternative approach to overcome the pharmacokinetic limitation of NTX, allowing reaching the alveolar spaces with high concentration comparing to the oral route [79,80]. While there is no approved inhaled antifungal for treating pulmonary fungal infections like invasive aspergillosis, results from preclinical and clinical studies for inhaled antifungals including azoles (voriconazole and itraconazole) supports the benefit of this approach in overcoming the PK limitations and reducing potential toxicities and drug-drug interactions [80–83].

In conclusion, this study elucidates various aspects of NTX activity against the *Cryptococcus* pathogen, including its modulation for the primary virulence traits of cryptococcal cells and its potential mechanism of action. Additionally, our study highlights the beneficial interaction between NTX and standard antifungals, especially FLC, and its efficacy in the nematode model of *C. elegans*, which necessitates further investigation in mammalian model to confirm its in vivo efficacy.

## Supporting information

S1 Raw Data In vitro evaluation of NTX efficacy. (XLSX)

S1 Fig Evaluation the efficacy of NTX/FLC combination in the *Caenorhabditis elegans* nematode model. **(A)** Impact of NTX on the fungal burden of infected *C. elegans* in combination with FLC. Infected L4 larvae (300 worms per mL) were individually treated with NTX at a sub-inhibitory concentration (0.5x MIC), FLC (2x MIC), or their combination for 24 hrs at 30°C. Treated worms were homogenized and plated onto gentamicin-supplemented YPD agar to assess the change in the fungal burden. Data was analyzed using one-way analysis of variance (ANOVA) for multiple comparison, and *p values* of ≤ 0.05 indicate statistical significance of compared data sets. **(B)** A Kaplan-Meier survival curve of *C. elegans* worm groups individually treated with NTX (0.5x MIC), FLC (2x MIC), or their combination. Statistical significance was evaluated using log-rank test.
(TIFF)

S2 Fig Growth of cryptococcal cells in the presence of a sublethal concentration of NTX or positive controls used in the virulence study. Inoculum of the *C. neoformans* H99 strain was adjusted to the exact density used for each virulence experiment and treated with NTX (0.5x MIC) and other positive controls including amphotericin B (AmB), acetohydroxamic acid (AHA), and flucytosine (5-FC). Following the end of each incubation time, aliquots of treated cryptococcal cells were serially diluted with phosphate-buffered saline and platted on YPD agar.
(TIFF)
